# Analysis of *Akkermansia muciniphila* in Mulberry Galacto-Oligosaccharide Medium via Comparative Transcriptomics

**DOI:** 10.3390/foods12030440

**Published:** 2023-01-17

**Authors:** Erna Li, Shipei Li, Fan Liu, Qian Li, Daorui Pang, Hong Wang, Sentai Liao, Yuxiao Zou

**Affiliations:** 1 Sericultural & Agri-Food Research Institute, Guangdong Academy of Agricultural Sciences/Key Laboratory of Functional Foods, Ministry of Agriculture and Rural Affairs/Guangdong Key Laboratory of Agricultural Products Processing, Guangzhou 510610, China; 2 College of Food Science, South China Agricultural University, Guangzhou 510642, China; 3 Sericultural & Agri-Food Research Institute, Guangdong Academy of Agricultural Sciences, No. 133 Yiheng St., Dongguanzhuang Rd., Tianhe District, Guangzhou 510610, China

**Keywords:** transcriptomics, *Akkermansia muciniphila*, mulberry galacto-oligosaccharide, mulberry polysaccharide, prebiotics

## Abstract

*Akkermansia muciniphila* is a common member of the human gut microbiota and belongs to the phylum Verrucomicrobia. Decreased levels of *A. muciniphila* are associated with many diseases, so it is thought to be a beneficial resident of the intestinal mucosal layer. In this study, we found that different prebiotics promoted the proliferation of *A. muciniphila*, and mulberry galacto-oligosaccharide (MGO) had the greatest effect. We cultured *A. muciniphila* in a brian heart infusion (BHI) medium containing 5% galactooligosaccharides (GOS), mulberry polysaccharide solution (MPS), and MGO, and transcriptomic analyses were performed. The results revealed that, after 6 days of cultivation, the numbers of upregulated functional genes (based on Gene Ontology) were approximately 0.7 and 19% higher with MPS and MGO, respectively, than with GOS. Analysis using the Kyoto Encyclopedia of Genes and Genomes showed that, when *A. muciniphila* was cultured with MGO, genes that were upregulated were enriched in the carbohydrate metabolism, the metabolism of cofactors and vitamins, the energy metabolism, the amino acid metabolism, and the lipid metabolism. Upregulated genes included *galM* and *pfkA* in the galactose metabolism, and *pgi*, *pfk*, *fbaA*, *tpiA*, *gapA*, *pgk*, *gpml*, *eno*, *pyk*, and *lpd* in the glycolysis/gluconeogenesis pathway. Real-time quantitative PCR results were consistent with the RNA-Seq data. This work provides valuable knowledge which can be available for the functional application of *A. muciniphila* and MGO.

## 1. Introduction

In recent years, research on the biological activities of plant functional polysaccharides has mainly focused on the regulation of sugar and lipid homeostasis, enhancing immunity, and improving the intestinal microbiota. Because plant functional polysaccharides have extremely low toxicity and few side effects, they are particularly widely used in the prevention and treatment of chronic diseases such as diabetes, obesity, and metabolic syndrome in humans [1,2]. Mulberry is widely cultivated in Asia, and it has various health benefits such as regulating sugar homeostasis, immune regulation, and acting as a laxative for humans [3,4]. Mulberry polysaccharides have been confirmed to play an important role in many biological activities of mulberry [5]. Chen et al. reported that mulberry polysaccharide has good antioxidant activity and can inhibit the absorption of glucose by inhibiting the activities of α-amylase and α-glucosidase in vitro [6]. Mulberry polysaccharide can upregulate the expression of the insulin receptor InsR, insulin substrate receptor IRS-2, and glucose transporter in type II diabetic rats and lower insulin resistance [7].

Polysaccharide can improve the proliferation rate of probiotic microbes in vitro. Huebner discovered that inulin, galactooligosaccharides, and fructooligosaccharides had proliferative effects on *Lactobacilli* and *Bifidobacterium*. Inulin can promote the highest proliferation rate of *Lactobacillus casei* strain 1195 [8]. We found that mulberry oligosaccharide prepared via the enzymatic hydrolysis of mulberry polysaccharide had a proliferative effect on *Lactobacillus rhamnosus* GG [9]. Increased probiotics in the body have many health effects, such as lowering blood sugar, improving obesity, and protecting the mucosal barrier of the gut [10,11]. Mulberry oligosaccharides can improve the glucose metabolism defect in type II diabetic mice by regulating the structure of the gut microbiota [12]. Based on current research, the effects of probiotics on general health are far from proven.

*Akkermansia muciniphila* (*A. muciniphila*) is an anaerobic Gram-negative bacterium that colonizes the mucus layer of the intestinal lumen. It has been isolated and cultured from human feces in recent years. It belongs to the phylum Verrucomicrobia and has a special function of degrading mammalian intestinal mucins [13]. The relationship between the bacterium and host metabolism is reflected not only in the energy intake, use, and consumption related to glucose, protein, and lipid metabolism, but also in the health of the mucus layer and the mucosal immune response [14]. *A. muciniphila* is significantly associated with type II diabetes. Populations with high abundances of *A. muciniphila* showed better metabolic statuses, especially in insulin sensitivity, fasting blood glucose, plasma triglycerides, and body fat distribution [15]. In type II diabetic mice, supplementation with oligofructose increased the amount of *A. muciniphila* in the gut and decreased insulin resistance [16]. Changes in the amount of *A. muciniphila* are important indications for the occurrence and development of diabetes. Ellekilde found that the content of *A. muciniphila* in average people is higher than that in prediabetic people. With the deterioration of glucose tolerance, the amount of *A. muciniphila* in the body further decreased or even disappeared, and the amount of *A. muciniphila* in the gut is negatively correlated with the occurrence of type II diabetes [17]. Everard administered *A. muciniphila* to obese and type II diabetic mice and found that high-fat-diet-induced metabolic disturbances, such as increased fat content, endotoxemia, adipose tissue inflammation, and insulin resistance, were decreased. This change may be achieved by increasing the level of endocannabinoids in the gut to regulate inflammation, the intestinal barrier, and intestinal peptide secretion [16].

In this study, we used transcriptomic analyses to explore the responses of *A. muciniphila* to the addition of different prebiotics to a BHI medium, and the underlying mechanism was interpreted. Real-time quantitative PCR experiments were performed on selected differential genes to verify the accuracy and repeatability of the RNA-seq data. Our findings provide a theoretical basis for the future development of mulberry prebiotics compounded with *A. muciniphila* as an intestinal prebiotic.

## 2. Materials and Methods

### 2.1. Chemicals and Strain

Galactooligosaccharides (GOSs) and isomaltooligosaccharides (IMOs) were purchased from Yuan-ye Co. (Shanghai, China). BHI medium and agar powder were purchased from Guangdong Huankai Microbial Sci. & Tech. Co., Ltd. (Guangzhou, China). Pectinase (1000 U/mg), glucoamylase (100 U/mg), xylanase (6000 U/mg), β-glucanase (50 U/mg), and α-amylase (3700 U/g) were purchased from Guangzhou Qiyun Biotechnology Co., Ltd. (Guangzhou, China). β-Mannanase (50 U/mg) was from Yuan-ye Co. (Shanghai, China).

*A. muciniphila* ATCC BAA-835 was purchased from the BeNa Culture Collection and stored in an anaerobic tube with 25% glycerol at −80 °C. It was a stationary culture for 3 days at 37 °C in 10 mL of BHI medium in a carbon dioxide incubator in an anaerobic chamber, to guarantee an oxygen-free environment.

### 2.2. Preparation, Isolation, and Purification of Mulberry Oligosaccharides

Mulberry polysaccharides (MPSs) were prepared following the method described by Chen et al. [18]. For the enzymatic hydrolysis of polysaccharides, MPSs were incubated with different individual enzymes (5%; pectinase, glucoamylase, β-mannanase, xylanase, β-glucanase, and α-amylase) at 50 °C for 4 h. Mulberry oligosaccharides (MOSs) produced from MPS via digestion with β-mannanase had the greatest effect on the proliferation of *A. muciniphila* in experiments. Mulberry oligosaccharides (MOSs) were prepared by incubating MPS with 5% (*w*/*v*) β-mannanase at 50 °C for 4 h, and then lyophilized.

MOSs were dissolved in water (10% *w*/*v*) and then loaded onto a DEAE-52 cellulose column (2.5 × 25 cm), previously equilibrated with water. The column was eluted with water and a step gradient of 0.1 and 0.3 mol/L NaCl at a flow-rate of 1 mL/min. The eluates were collected with an automatic collector (10 mL in one tube). The elution profile was detected using the phenol-sulfuric acid assay. It could be divided into three main elution peaks, namely MOS-I, MOS-II, and MOS-III, which were then lyophilized. MOS-I was selected for further fractionation because of its higher proliferation rate on *A. muciniphila* proliferation. Size-exclusion chromatography on a Sephadex G-100 column (2.5 × 25 cm) with water at a flow-rate of 1 mL/min yielded a fraction which was named MOS-Ia and then lyophilized. On the basis of previous results, the purified oligosaccharide was composed of galactose. The average molecular weight was 987 Da [19]. Because it solely contains galactose, it was named mulberry galacto-oligosaccharide (MGO).

The number of colony-forming units of colonies of *A. muciniphila* in BHI agar medium with no added oligosaccharide was defined as having a proliferation level of 100%, where the proliferation level was calculated as:

$$\mathrm{Proliferation}\;\mathrm{level}=\frac{\mathrm{The}\;\mathrm{number}\;\mathrm{of}\;\mathrm{colonies}\;\mathrm{in}\;\mathrm{BHI}\;\mathrm{agar}\;\mathrm{medium}\;\mathrm{with}\;\mathrm{oligosaccharide}}{\mathrm{The}\;\mathrm{number}\;\mathrm{of}\;\mathrm{colonies}\;\mathrm{in}\;\mathrm{BHI}\;\mathrm{agar}\;\mathrm{medium}\;\mathrm{with}\;\mathrm{no}\;\mathrm{oligosaccharide}}\times 100\%$$

### 2.3. Effects of Different Prebiotics on the Growth of A. muciniphila

The positive control group was the GOS group. The negative control group was medium without any added oligosaccharide. GOS, MPS, or MGO were added to BHI medium to achieve the concentration at 5% (*w*/*v*). Then, the medium was anaerobically cultured and inoculated with 5% (*v*/*v*) of culture of *A. muciniphila* for 5 days. Samples were plated every day to calculate the number of colonies.

### 2.4. RNA-Seq and Real-Time Quantitative PCR (RT-qPCR) Validation

#### 2.4.1. Bioinformatic and Differential Expression Analyses

The maximum biomass stage of *A. muciniphila* growth (after 6 days of anaerobic culture) was selected as the sampling time point for comparative transcriptomics. There were three replicates per group. A total RNA extractor extracted the total RNA following the method described by Li et al. [9]. RNA quality was determined with a 2100 Bioanalyser (Agilent Technologies Co., Ltd., Colorado Springs, CO, USA) and quantified using an ND-2000 (NanoDrop Technologies/Thermo Scientific, Wilmington, DE, USA). An RNA-Seq transcriptome library was prepared with the TruSeq RNA Sample Preparation Kit from Illumina (San Diego, CA, USA) using 2 μg of total RNA. Shortly after preparation, ribosomal (r)RNA depletion was performed using the Ribo-Zero Magnetic Kit (Epicenter); then, all mRNAs were broken into short fragments by adding fragmentation buffer. The data generated by the Illumina platform would be used to analyze bioinformatics. The data were analyzed using the online platform Majorbio Cloud Platform (www.Majorbo. com (accessed on 3 September 2021)) at Shanghai Majorbio Bio-pharm Technology Co., Ltd., Shanghai China. For each data set, and for each alignment and quantification protocol, we identified differentially expressed genes (DEGs) by using the edgeR, DESeq2, and DESeq packages.

#### 2.4.2. Gene Ontology (GO) and Kyoto Encyclopedia of Genes and Genomes (KEGG) Enrichment Analysis

DEGs between the control group (BHI medium group) and treatment groups (5% (*w*/*v*) GOS added to the BHI medium group, 5% (*w*/*v*) MPS added to the BHI medium group, and 5% (*w*/*v*) MGO added to BHI medium group) were assigned using GO and KEGG.

#### 2.4.3. Validation of RNA-Seq Results by RT-qPCR

Thirteen DEGs associated with the galactose metabolism (*galM*, *pfkA*, and *pgm*) or glycolysis/gluconeogenesis (*pgi*, *pfk*, *fbaA*, *tpiA*, *gapA*, *pgk*, *gpml*, *eno*, *pyk*, and *lpd*) were employed to validate the RNA-seq results. Gene names and primer sequences used for RT-qPCR are shown in Table 1. The PCR programs included 40 cycles of 95 °C for 5 s, annealing at 55 °C for 30 s, and extension at 72 °C for 40 s. The 16S rRNA gene was an internal standard to normalize gene expression. Three independent repetitions were performed for each sample, and the 2^−ΔΔCt^ method was used to calculate the relative expression levels of genes.

### 2.5. Statistical Analysis

All experiments were represented as the mean ± standard deviation of three replicated measurements, and the results were analyzed using SPSS-19 software (Chicago, IL, USA). Statistical significance (*p* < 0.05) between treatments was analyzed using one-way analysis of variance, followed by Duncan’s multiple-range test.

## 3. Results and Discussion

### 3.1. Proliferation of A. muciniphila Cultured with Different Carbohydrates

In this study, MOS was produced from MPS via digestion with different kinds of enzymes (pectinase, glucoamylase, β-mannanase, xylanase, β-glucanase, and α-amylase). Using commercial prebiotics (GOS and IMO) as controls, the effects of mulberry oligosaccharides with different concentrations on the proliferation of *A. muciniphila* were investigated (Table 2). It was found that the effect of mulberry oligosaccharide prepared by adding 5% β-mannanase was the greatest on the proliferation of *A. muciniphila*. The proliferation of the bacterium following treatment with mulberry oligosaccharide produced using 5% β-mannanase (652% ± 9.9%) was superior to that following treatment with GOS (582% ± 10.2%). We speculate that the composition of the sugar chain after enzymatic hydrolysis is an important factor affecting the proliferation of *A. muciniphila*.

**Figure 1:**
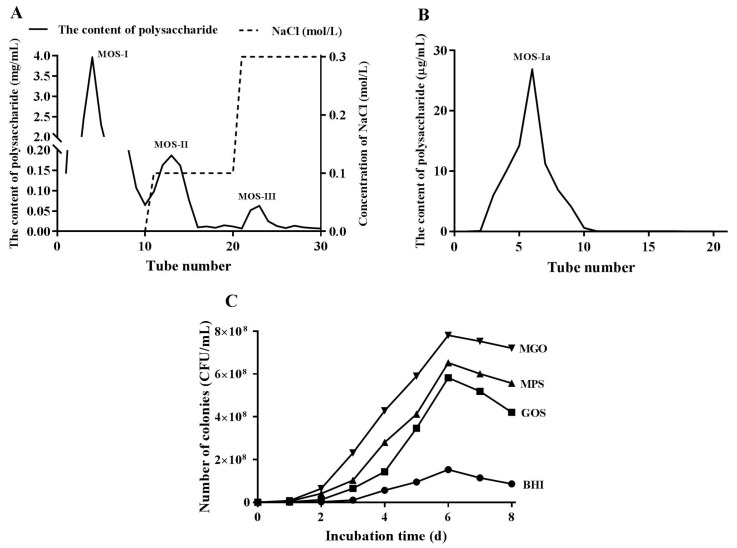
Elution profile of MOS produced via digestion of MPS 5% β-mannanase from a DEAE-52 cellulose column (**A**). Elution of fraction MOS-I from a Sephadex G-100 chromatography column (**B**). Curve of *Akkermansia muciniphila* growth in BHI medium supplemented with different prebiotics (**C**).

The mulberry oligosaccharides prepared using β-mannanase were separated into three fractions—MOS-1, MOS-2, and MOS-3 via DEAE-cellulose column chromatography (Figure 1A). When adding 5% (*w*/*v*) MOS-1, the proliferation level of *A. muciniphila* was 781% ± 10.5 (Table 3). MOS-I was further separated via Sephadex G-100 chromatography in distilled water (Figure 1B). A fraction termed MOS-Ia was obtained, which showed a single peak in the chromatogram. On the basis of our previous research, MOS-Ia is composed of galactose. The average molecular weight was 987 Da. We named it mulberry galactooligosaccharide (MGO), because the oligosaccharide only contains galactose units [19]. Figure 1C shows the growth curves of *A. muciniphila* in different BHI media when adding 5% (*w*/*v*) GOS, MPS, or MGO. When cultured for 6 days, the total number of colonies reached a maximum value of about 7.8×10^8^ colony-forming units (CFU)/mL with the addition of 5% (*w*/*v*) MGO. The number of CFU increased about by fivefold in MGO medium compared with the control (BHI with no added prebiotics, 1.5 × 10^8^ CFU/mL). Since the total number of *A. muciniphila* colonies reached the maximum value on the sixth day, the following transcriptome sequencing experiments were performed after 6 days of culture.

The degree of polymerization affects the proliferation of probiotics caused by prebiotics. Prebiotics with a low degree of polymerization are more readily available to probiotics. Compared with MPS, IMO, or GOS, it was found that MGO had the lowest degree of polymerization, which had a greater proliferative effect on *A. muciniphila*. In addition to the degree of polymerization, the chemical structure, monosaccharide composition, degree of branching, and water solubility have an impact on the prebiotic effects. Therefore, a simple chemical structure, high water solubility, and more branches ending in carbohydrate chains in oligosaccharides are considered to have better proliferative effects on probiotics.

### 3.2. Differential Gene Expression for Analysis

#### 3.2.1. Cluster Analysis of Differential Gene Expression

**Figure 2:**
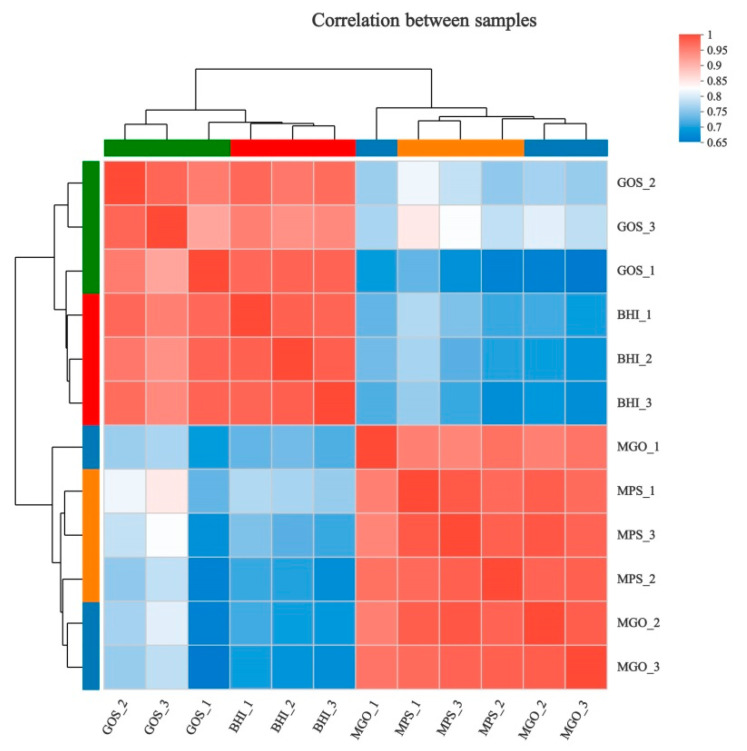
Clustering analysis of differentially expressed genes (DEGs) in culture of *A. muciniphila* with different prebiotics.

The Pearson correlation coefficient is shown in Figure 2. The higher the intensity of the red color, the higher the correlation between two samples and a smaller difference between them. The biological duplication from each group had good repeatability, and the group composed of BHI and GOS was significantly different from the groups composed of MPS and MGO.

#### 3.2.2. Identification of DEGs

**Figure 3:**
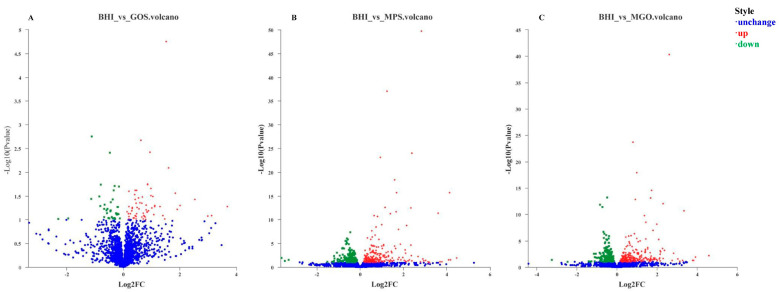
Volcano plots of DEGs in *A. muciniphila* after different prebiotic treatments. Volcano plot of DEGs in GOS (**A**); volcano plot of DEGs in MPS (**B**); volcano plot of DEGs in MGO (**C**).

In order to clarify the gene expression responses of *A. muciniphila* in different prebiotic treatments, transcriptomic analysis was carried out to identify the DEGs after 6 days of culture. A total of 1277, 1216, and 1240 genes were upregulated when GOS, MPS, and MGO were added to the BHI medium, respectively. Furthermore, 1150, 1205, and 1185 genes were shown to be downregulated. In the respective media, the expression of 159, 165, and 161 genes did not change. Statistical analyses are shown in volcano plots in Figure 3.

As shown in supplementary excel 1, 119, 195, and 187 genes were upregulated when GOS, MPS, or MGO was added to the BHI medium, with a more than 2 log2-fold change. Genes *AMUC_RS05445*, *AMUC_RS06065*, *AMUC_RS07280*, *AMUC_RS07405*, *AMUC_RS08975*, and *AMUC_RS09930*, which, respectively, encode a hypothetical protein, M15 family metallopeptidase, DUF1778 domain-containing protein, acyltransferase, nicotinate-nucleotide-dimethylbenzimidazole phosphoribosyltransferase, and DUF2075 domain-containing protein, were highly expressed. The M15 family metallopeptidase of the zinc-binding metallopeptidase family, which contains mostly carboxypeptidases and dipeptidases, is involved in bacterial cell wall biosynthesis and metabolism [20]. Acyltransferase A was found to only have affinity for short-chain aliphatic amides with maximum activity towards acetamide [21]. Nicotinate-nucleotide–dimethylbenzimidazole phosphoribosyltransferase catalyzes the synthesis of alpha-ribazole-5′-phosphate from nicotinate mononucleotide and 5,6-dimethylbenzimidazole [22].

### 3.3. GO Annotation Analysis of DEGs

**Figure 4:**
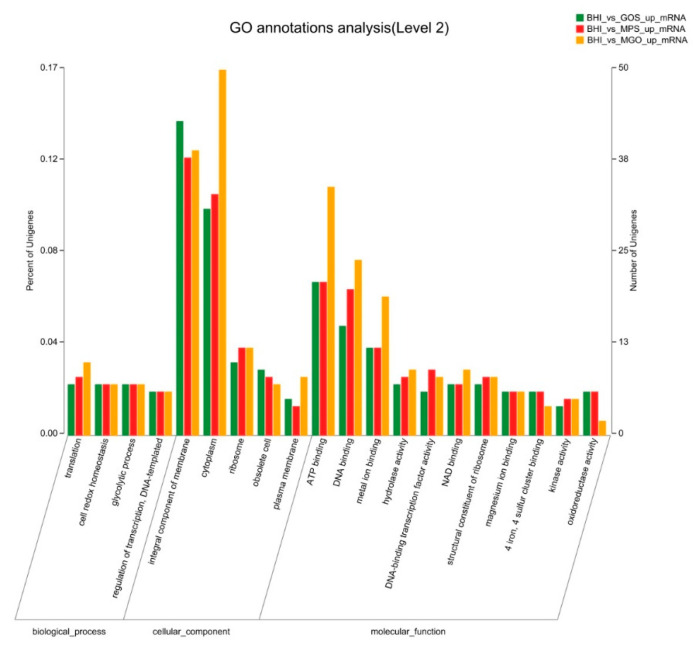
Gene Ontology (GO) analysis of *A. muciniphila* cultured with different prebiotics for 6 days. The right *y*-axis represents the number of unigenes of a specific category within the main category, and the left *y*-axis represents the percentage of unigenes of a specific category within the main category. A bar indicates the number of upregulated genes.

To clarify the changes that occur in biological processes of *A. muciniphila* on treatment with different prebiotics, GO term enrichment analysis was performed on the up- and downregulated genes identified via RNA-Seq analysis. As shown in Figure 4, upregulated GO functional gene enrichment analysis resulted in a list of affected biological processes, cellular components, and molecular functions. The molecular function category contained the greater part of the GO annotations, followed by the biological process and cellular component categories. In molecular function, the main affected categories were ATP binding, DNA binding, and metal ion binding. In the cellular component ontology, most DEGs were associated with the integral components of membrane, cytoplasm, and ribosome. Among the biological process, most enriched DEGs were in translation, cell redox homeostasis, and glycolytic processes. The upregulated GO functional gene component categories for the GOS, MPS, and MGO treatments contained 556, 560, and 659 genes, respectively; that is, the value was highest when MGO was added to *A. muciniphila* cultures.

### 3.4. KEGG Analysis of DEGs

**Figure 5:**
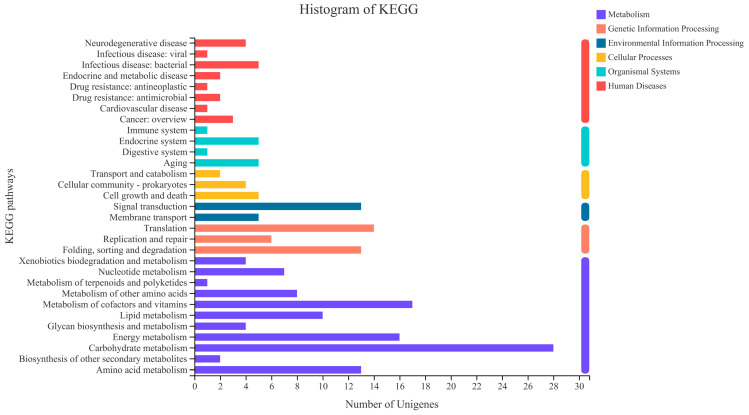
Comparative transcriptomic analyses of *A. muciniphila* cultured with MGO for 6 days. KEGG pathway enrichment analysis of DEGs. The horizontal coordinates represent the KEGG pathway name, and the vertical coordinates represent the number of transcript/metabolite unigenes.

To further identify changes in biochemical pathways following different prebiotic treatments, we used the KEGG database to map DEGs [23]. KEGG pathways containing DEGs were identified for analysis (Figure 5). Among them, the maximum number of DEGs of five pathways when the BHI medium was cultured with MGO were the carbohydrate metabolism, the metabolism of cofactors and vitamins, the energy metabolism, the amino acid metabolism, and the lipid metabolism.

MGO is an oligosaccharide which only contains galactose units. Combined with the KEGG pathology analysis results, we supposed that the MGO-dependent proliferation mechanism of *A. muciniphila* stimulates genetic changes which correlate with the carbohydrate metabolism, especially the galactose metabolism and glycolysis/gluconeogenesis. Thus, in order to understand the molecular mechanisms underlying the effects of MGO on the galactose metabolism and glycolysis/gluconeogenesis, we screened DEGs related to these two pathways in the transcriptomic sequencing results (Table 4).

**Figure 6:**
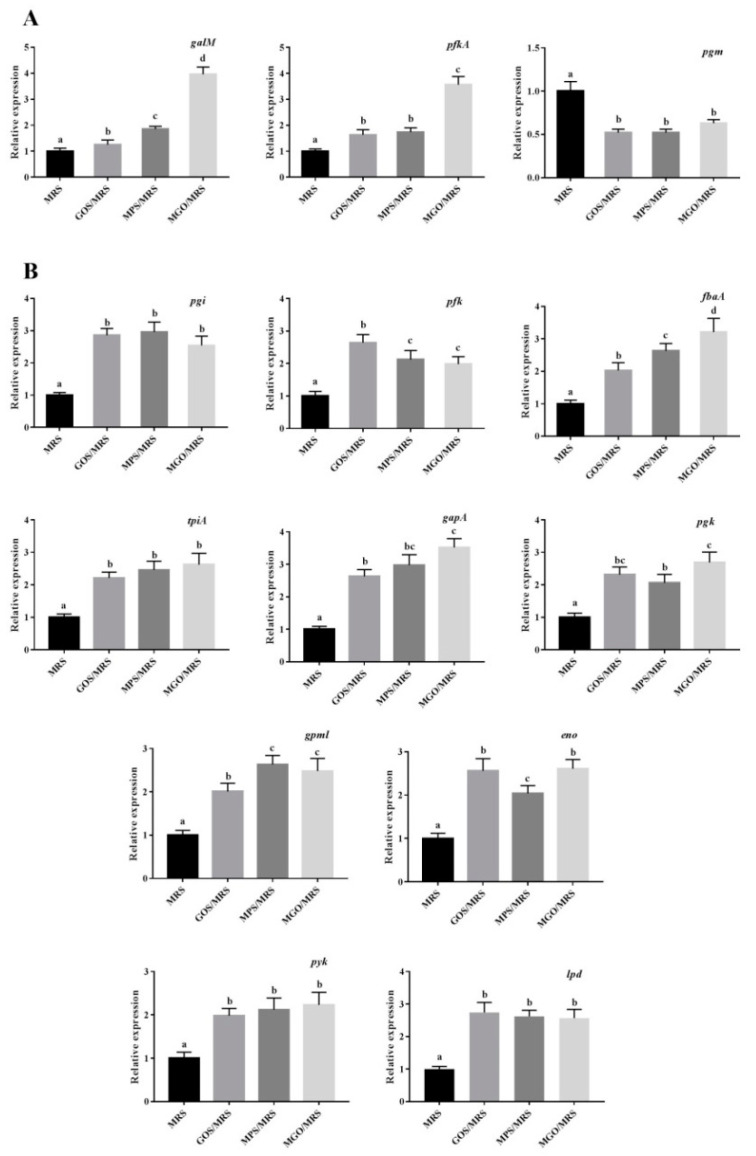
The qRT-PCR results to validate the transcriptions of galactose-metabolism-pathway-related genes (**A**) and glycolysis/gluconeogenesis-pathway-related genes (**B**) mentioned in RNA-seq data.

Two DEGs (*galM* and *pfkA*) were upregulated and one (*pgm*) was downregulated in relation to the galactose metabolism. RT-qPCR data (Figure 6A) were almost the same as the gene expression obtained from RNA-Seq, indicating that the data from the RNA-seq were reliable. In RT-qPCR, the mRNA levels of *galM* and *pfkA* increased by 396% and 357%, respectively, and that of *pgm* was decreased to 63% when the addition of MGO into the BHI medium reached 4% (*w*/*v*).

In the KEGG, *galM* is assigned as galactose mutarotase. Galactose mutarotase is an important metabolism-related enzyme in bacteria which catalyzes the change in the optical rotation of α-D-galactose to generate β-D-galactose, the only substrate of galactose kinase. Galactose mutarotase is thus a key enzyme in the process of galactose metabolism and an important member of the galactose operon [24,25,26,27]. The KEGG pathway assignment of *pfkA* is 6-phosphofructokinase. The 6-phosphofructokinase enzyme is the rate-limiting enzyme of the glycolytic pathway; its activity strictly controls the rate of glycolysis and greatly affects the use of hexoses by bacteria. The 6-phosphofructokinase enzyme is a key node in the glucose metabolism, which is accompanied by a large amount of energy consumption. The loss of 6-phosphofructokinase leads to an imbalance of reducing power, which affects downstream metabolic pathways, including pathways related to nitrogen fixation. Therefore, in bacteria, it directly affects the use of carbon sources [28,29,30,31].

Eleven DEGs (*pgi*, *pfk*, *fbaA*, *tpiA*, *gapA*, *pgk*, *gpml*, *eno*, *pyk*, and *lpd*) involved in glycolysis/gluconeogenesis were upregulated, and one (*pgm*) was downregulated when the addition of MGO into the BHI medium reached 4% (*w*/*v*) (Figure 6B). The DEGs were proven via RT-qPCR, which showed that *pgi*, *pfk*, *fbaA*, *tpiA*, *gapA*, *pgk*, *gpml*, *eno*, *pyk*, and *lpd* expression was promoted by 254, 198, 321, 262, 352, 269, 248, 261, 223, and 257% when *A. muciniphila* was grown in BHI medium with 4% (*w*/*v*) MGO.

The *pgi* in KEGG pathway is glucose-6-phosphate isomerase. Glucose-6-phosphate isomerase exists in eukaryotes and prokaryotes, and it is a multifunctional enzyme whose main function is to catalyze the mutual conversion between glucose-6-phosphate and fructose-6-phosphate in the process of glycolysis [32,33,34,35,36]. *tpiA* encodes triose-phosphate isomerase, an enzyme in the glycolytic pathway. In this pathway, 1,6-diphosphate fructose reacts with aldolase to generate dihydroxyacetone phosphate and glyceraldehyde 3-phosphate. Dihydroxyacetone phosphate and glyceraldehyde 3-phosphate can be interconverted under the catalysis of triose-phosphate isomerase; glyceraldehyde 3-phosphate will continue to be catalyzed to pyruvic acid [37,38,39]. The KEGG pathway assignment of *eno* is phosphopyruvate hydratase. Phosphopyruvate hydratase is a key enzyme in the glucose metabolism pathway and is expressed abundantly in the cytoplasm of many organs. Its role is to convert 2-phospho-glycerate into phosphoenolpyruvate [40,41]. The KEGG pathway assignment of *pyk* is pyruvate kinase. Pyruvate kinase is involved in the last reaction of glycolysis and is one of the main rate-limiting enzymes in the process [42]. It catalyzes the synthesis of one molecule of adenosine triphosphate and pyruvate from adenosine diphosphate and phosphoenolpyruvic acid, which requires the participation of Mg^2+^ [43]. Pyruvate kinase is produced in all cells and tissues capable of glycolysis, and its metabolites, adenosine triphosphate and pyruvate, are used in numerous biosynthetic pathways [44,45]. Pyruvate kinase is a master regulator that controls adenosine triphosphate production in glycolysis and is considered a potential drug target [46,47]. However, because of the conserved structure of the active site of pyruvate kinase and its central role in glycolysis in all organisms, the development of selective inhibitors against the active site of this enzyme has been difficult.

## 4. Conclusions

In the present study, MGO was found to be a good stimulus to proliferate *A. muciniphila*. We discovered that after adding MGO to the BHI medium, GO functional analysis revealed that 659 genes were upregulated in *A. muciniphila*. These genes were mainly enriched in ATP binding, an integral component of membranes, and translation. When DEGs were mapped to the KEGG database, the upregulated genes were enriched in the carbohydrate metabolism, the metabolism of cofactors and vitamins, the energy metabolism, the amino acid metabolism, and the lipid metabolism. In the significantly up-regulated genes, *galM* and *pfkA* are relevant to the galactose metabolism, and *pgi*, *pfk*, *fbaA*, *tpiA*, *gapA*, *pgk*, *gpml*, *eno*, *pyk*, and *lpd* are relevant to the glycolysis/gluconeogenesis pathway. Our research provides a more in-depth theoretical basis for the application of *A. muciniphila* and MGO.

## Figures and Tables

**Table 1:** List of primers for RT-qPCR.

Gene	Forward Primer (5′–3′)	Reverse Primer (5′–3′)
AMUC_RS07000 (*galM*)	CCTCACCAACCACGCTTACT	TATAGGCGGAGGCCCGTATC
AMUC_RS01180 (*pfkA*)	TATTCCGGCCACGATTGACA	GCCGTATCACGAACGGAATC
AMUC_RS00900 (*pgm*)	TGAAGTGGTCAACGTGCTCA	GCGTTTGTTCCTCGCTCATC
AMUC_RS10555 (*pgi*)	AGTAAGCGTGGTTGGTGAGG	AAACGCCCTGTCTATCCGTC
AMUC_RS07935 (*pfk*)	TTATGCCGTGGAACTGGTGG	TTCCTCAATGGGTACGGCAG
AMUC_RS03915 (*fbaA*)	TAGGCAATTCCGCCCTTGG	TGTGAAGGGCTACGAGAACG
AMUC_RS03080 (*tpiA*)	TGGAACCCGTGCTGGAAATC	TAGGCGATGACCAGGTTGGA
AMUC_RS07575 (*gapA*)	TTGCTCCGATGGTGAAGGTG	GCTGGTCGTTCGTGTAGGAG
AMUC_RS07580 (*pgk*)	TGAAATGGACTGCTTCGCCA	CGCCGCCTACAATGGAAATG
AMUC_RS01755 (*gpml*)	TGTGGAGCAGTGTTATGCGA	TTATCCCTCACGCGCTGTTC
AMUC_RS04575 (*eno*)	CTGGAAGCCACGGAACAAAC	AAACACCCAGAATGGCGTTG
AMUC_RS02385 (*pyk*)	AAACGCCTGTCTGATCGTCT	GGTCATTGCTGAAGGCGAAG
AMUC_RS09020 (*lpd*)	CTACTGTCATCGGCTCCAGG	AATTCCGTGCCGATAGCTCC
AMUC _16SrRNA	AAGGGTTTCGGCTCGTAAAA	TGCACTCAAGTTTCCCAGTT

**Table 2:** Proliferation of *A. muciniphila* cultured in medium containing different prebiotic carbohydrates after 5 days of incubation at 37 °C.

Carbohydrate	Concentration (%)	*A. muciniphila* Proliferation
GOS	1	178% ± 8.3a
	3	269% ± 7.5b
	5	582% ± 10.2c
IMO	1	156% ± 5.3a
	3	204% ± 9.4b
	5	351% ± 11.2c
MPS	1	159% ± 5.4a
	3	208% ± 6.8b
	5	256% ± 7.2c
MPS hydrolyzed via pectinase	1	151% ± 4.3a
	3	201% ± 7.3b
	5	330% ± 9.5c
MPS hydrolyzed via glucoamylase	1	185% ± 3.5a
	3	241% ± 6.3b
	5	484% ± 8.9c
MPS hydrolyzed via β-mannanase	1	202%± 5.4a
	3	405% ± 7.2b
	5	652% ± 9.9c
MPS hydrolyzed via xylanase	1	103% ± 2.3a
	3	135% ± 4.4b
	5	198% ± 5.2c
MPS hydrolyzed via β-glucanase	1	158% ± 3.9a
	3	221% ± 4.5b
	5	412% ± 5.1c
MPS hydrolyzed via α-amylase	1	109% ± 2.2a
	3	125% ± 3.1b
	5	132% ± 2.8c

The data are presented as the mean ± SD of three replicates. Means marked with the same letter are not significantly different (*p* < 0.05) according to Duncan’s multiple range test. Abbreviations: MPS, crude mulberry polysaccharide solution.

**Table 3:** Proliferation of *A. muciniphila* cultured in medium containing different elution fractions of mulberry oligosaccharides prepared using β-mannanase after incubation for 5 days at 37 °C.

Carbohydrate	Concentration (%)	*A. muciniphila* Proliferation
MOS-I	1	254% ± 3.2a
	3	497% ± 5.8b
	5	781% ± 10.5c
MOS-II	1	125% ± 4.2a
	3	198% ± 3.5b
	5	245% ± 6.8c
MOS-III	1	132% ± 2.3a
	3	201% ± 4.8b
	5	253% ± 6.1c

The data are presented as the mean ± SD of three replicates. Means marked with the same letter are not significantly different (*p* < 0.05) according to Duncan’s multiple range test.

**Table 4:** Effect of different prebiotics on gene transcription in *A. muciniphila* cultured for 6 days.

Gene ID (KEGG Name)	Gene Description	FC			Style
		GOS/BHI	MPS/BHI	MGO/BHI	
galactose metabolism					
AMUC_RS07000 (*galM*)	galactose mutarotase	1.18	1.36	3.47	up
AMUC_RS01180 (*pfkA*)	6-phosphofructokinase	1.30	1.32	3.16	up
AMUC_RS00900 (*pgm*)	phospho-sugar mutase	0.42	0.62	0.52	down
glycolysis/gluconeogenesis					
AMUC_RS07000 (*galM*)	galactose mutarotase	1.18	1.36	3.47	up
AMUC_RS10555 (*pgi*)	glucose-6-phosphate isomerase	2.24	2.52	2.31	up
AMUC_RS07935 (*pfk*)	6-phosphofructokinase	2.34	1.85	1.68	up
AMUC_RS03915 (*fbaA*)	class II fructose-bisphosphate aldolase	1.87	2.21	2.85	up
AMUC_RS03080 (*tpiA*)	triose-phosphate isomerase	1.98	2.12	1.87	up
AMUC_RS07575 (*gapA*)	type I glyceraldehyde-3-phosphate dehydrogenase	2.12	2.54	2.74	up
AMUC_RS07580 (*pgk*)	phosphoglycerate kinase	2.01	1.84	2.32	up
AMUC_RS01755 (*gpml*)	2%2C3-bisphosphoglycerate-independent phosphoglycerate mutase	1.87	2.31	2.14	up
AMUC_RS04575 (*eno*)	phosphopyruvate hydratase	2.24	1.89	2.18	up
AMUC_RS02385 (*pyk*)	pyruvate kinase	1.63	1.85	1.77	up
AMUC_RS09020 (*lpd*)	dihydrolipoyl dehydrogenase	2.32	2.11	2.25	up
AMUC_RS00900 (*pgm*)	phospho-sugar mutase	0.56	0.49	0.47	down

## Data Availability

The data are available from the corresponding author.

## Supplementary Materials

The following supporting information can be downloaded at: https://www.mdpi.com/article/10.3390/foods12030440/s1, Table S1: DEGs in *A. muciniphila* cultured for 6 days in different media.
